# Difference in Membrane Repair Capacity Between Cancer Cell Lines and a Normal Cell Line

**DOI:** 10.1007/s00232-016-9910-5

**Published:** 2016-06-16

**Authors:** Stine Krog Frandsen, Anna K. McNeil, Ivana Novak, Paul L. McNeil, Julie Gehl

**Affiliations:** Center for Experimental Drug and Gene Electrotransfer, Department of Oncology, Herlev and Gentofte Hospital, University of Copenhagen, Herlev, Denmark; Department of Cellular Biology and Anatomy, Georgia Regents University, Augusta, GA USA; Section for Cell Biology and Physiology, Department of Biology, University of Copenhagen, Copenhagen, Denmark

**Keywords:** Membrane repair, Electroporation, Cancer, Normal, In vitro

## Abstract

Electroporation-based treatments and other therapies that permeabilize the plasma membrane have been shown to be more devastating to malignant cells than to normal cells. In this study, we asked if a difference in repair capacity could explain this observed difference in sensitivity. Membrane repair was investigated by disrupting the plasma membrane using laser followed by monitoring fluorescent dye entry over time in seven cancer cell lines, an immortalized cell line, and a normal primary cell line. The kinetics of repair in living cells can be directly recorded using this technique, providing a sensitive index of repair capacity. The normal primary cell line of all tested cell lines exhibited the slowest rate of dye entry after laser disruption and lowest level of dye uptake. Significantly, more rapid dye uptake and a higher total level of dye uptake occurred in six of the seven tested cancer cell lines (*p* < 0.05) as well as the immortalized cell line (*p* < 0.001). This difference in sensitivity was also observed when a viability assay was performed one day after plasma membrane permeabilization by electroporation. Viability in the primary normal cell line (98 % viable cells) was higher than in the three tested cancer cell lines (81–88 % viable cells). These data suggest more effective membrane repair in normal, primary cells and supplement previous explanations why electroporation-based therapies and other therapies permeabilizing the plasma membrane are more effective on malignant cells compared to normal cells in cancer treatment.

## Introduction

**Fig. 1:**
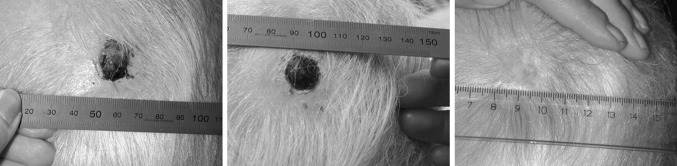
Malignant melanoma treated with electrochemotherapy. Pictures of a malignant melanoma patient before (*left*), 1 month (*middle*), and 6 months (*right*) after treatment with electrochemotherapy using bleomycin showing complete remission of the tumor 6 months after treatment. Note the needle marks seen in surrounding normal tissue 1 month after treatment showing that normal tissue is much less affected by electrochemotherapy then the cancer tissue (Gehl 2005)

Electroporation is increasingly being used in cancer treatment strategies (Kee et al. 2011). It is a method where application of short, high-voltage pulses induces transient permeabilization of the cell membrane and thus allows the passage of ions and molecules into and out of the cell (Orlowski and Mir 1993; Gehl 2003; Frandsen et al. 2012; Vasquez et al. 2015). The membrane reseals within a few minutes depending on the parameters used (reversible electroporation) and if the electric field is high enough, the membrane does not reseal and results in irreversible electroporation (Rols and Teissie 1990). Clinically, in anti-cancer treatments, this method is used as irreversible electroporation (without added drugs) (Martin et al. 2015) and as reversible electroporation in combination with chemotherapeutic drugs (electrochemotherapy) (Belehradek et al. 1993; Marty et al. 2006; Matthiessen et al. 2012), calcium (calcium electroporation) (ClinicalTrials.gov ID- NCT01941901), and DNA drugs (gene electrotransfer) (Mir et al. 1999; Daud et al. 2008; Spanggaard et al. 2013). Electrochemotherapy is standardly used for the treatment of cutaneous metastases, and clinical trials for the treatment of internal tumors are ongoing using new electrode designs (Edhemovic et al. 2011). Interestingly, it has been a consistent clinical observation that normal tissue is much less affected than malignant tissues when treating with electrochemotherapy (Fig. 1; Gehl 2005) as well as with irreversible electroporation (Neal et al. 2011). Recently, similar results for calcium electroporation were shown in a 3D in vitro model where normal cell spheroids were much less affected by calcium electroporation than cancer cell spheroids (Frandsen et al. 2015). It has also been shown in vitro that sonoporation, a method where application of low-power ultrasound permeabilizes the cell membrane, causes different effects in normal and malignant cells (Lejbkowicz et al. 1993; Lejbkowicz and Salzberg 1997).

These differences in sensitivity between normal and cancer cells when permeabilizing the plasma membrane, independent of the method used, could indicate a difference in susceptibility of cells to permeabilization treatments or the capacity to rapidly reseal after permeabilization. We aimed to investigate if differences in membrane repair in a number of cancer cell lines, an immortalized cell line, and a normal primary cell line could be part of the explanation for the observed difference in sensitivity between normal and cancer cells for treatments using permeabilization methods. The extent of permeabilization after electroporation is known to depend on different factors including membrane composition (Levine and Vernier 2012), cell size (Teissie and Rols 1993), cell shape (Pucihar et al. 2006), and cell density (Pucihar et al. 2007) in the suspension. To our knowledge the observed difference in sensitivity between malignant and normal cells has been an empirical finding, for which mechanisms still need to be elucidated. We therefore decided to investigate membrane repair by disrupting the plasma membrane using laser technology, since holes created by laser, in contrast to permeabilization after electroporation, are equal in size independent of cell type (Bansal et al. 2003; Howard et al. 2011a, b), allowing us to investigate membrane repair separately from the level of membrane poration. We show that when using a laser to disrupt the plasma membrane in the presence of a fluorescent dye, the rate and extent of dye entry in the normal dermal fibroblast was the lowest in all tested cell lines and significantly reduced compared to that in six of the seven tumor-derived cell lines as well as the immortalized cell line. Thus, the normal fibroblast cell line appeared to have a faster membrane repair. These empiric observations could help explain the differential sensitivity to electrochemotherapy and calcium electroporation between normal and malignant cells.

## Materials and Methods

This was a collaborative study where the laser experiments were performed in Georgia, USA and the electroporation experiments were performed in Copenhagen, Denmark.

### Cell Culture

Nine different human cell lines were used in this study; seven cancer cell lines, an immortalized normal cell line, and one primary normal cell line (Table 1). (1) H69, a small cell lung carcinoma kindly provided by the Department of Radiation Biology, Copenhagen University Hospital, Denmark (Gjetting et al. 2010), (2) HT29, a colorectal adenocarcinoma (ATCC #HTB-38), (3) MKN-28, a gastric adenocarcinoma and (4) MKN-45, a gastric carcinoma kindly provided by Dr. Katsuya Miyake, Kagawa University, Japan (Fukui et al. 2003), and (5) PC3-M, a prostate cancer derived from a bone metastasis (ATCC #CRL-1435). These cell lines were all grown in RPMI-1640 culture medium (Gibco, Invitrogen). (6) MRC5, an immortalized lung fibroblast (ATCC #CCL-171) was grown in EMEM culture medium (Gibco, Invitrogen). (7) MDA-MB-231, a breast adenocarcinoma (ATCC #HTB-26), (8) SW780, a bladder transitional cell carcinoma kindly provided by Dr. Lars Dyrskjøt Andersen, Department of Molecular Medicine, Aarhus University Hospital, Skejby, Denmark (Herbsleb et al. 2008), and (9) primary normal human dermal fibroblasts HDF-n kindly provided by Dr. Marie-Pierre Rols, Institute of Pharmacology and Structural Biology, IPBS, Toulouse, France (Frandsen et al. 2015) were grown in DMEM culture medium (Gibco, Invitrogen). All cells grew with 10 % fetal calf serum (Gibco, Invitrogen), 100 U/ml penicillin, and 100 µg/ml streptomycin and were maintained at 37 °C and 5 % CO_2_. All cells were tested negative for mycoplasma using MycoAlert mycoplasma detection kit (Lonza).

**Table 1:** Cell lines used in this study

Cell line	Phenotype characteristics	Source	References
H69	Human small cell lung carcinoma	Kindly provided by the Department of Radiation Biology, Copenhagen University Hospital, Denmark	Gjetting et al. (2010)
HDF-n	Human primary normal human dermal fibroblasts (not immortalized)	Kindly provided by Dr. Marie-Pierre Rols, Institute of Pharmacology and Structural Biology, IPBS, Toulouse, France	Frandsen et al. (2015)
HT29	Human colorectal adenocarcinoma	ATCC #HTB-38	http://atcc.org/Products/All/HTB-38.aspx
MDA-MB-231	Human breast adenocarcinoma	ATCC #HTB-26	http://atcc.org/Products/All/HTB-26.aspx
MKN-28	Human gastric adenocarcinoma	Kindly provided by Dr. Katsuya Miyake, Kagawa University, Japan	Fukui et al. (2003)
MKN-45	Human gastric carcinoma	Kindly provided by Dr. Katsuya Miyake, Kagawa University, Japan	Fukui et al. (2003)
MRC5	Immortalized human lung fibroblast	ATCC #CCL-171	http://atcc.org/Products/All/CCL-171.aspx
PC3-M	Human prostate cancer derived from a bone metastasis	ATCC #CRL-1435	http://atcc.org/Products/All/CRL-1435.aspx
SW780	Human bladder transitional cell carcinoma	Kindly provided by Dr. Lars Dyrskjøt Andersen, Department of Molecular Medicine, Aarhus University Hospital, Skejby, Denmark	Herbsleb et al. (2008)

The name, phenotype characteristics, source, and references of the nine different cell lines used in this study are presented in the table

### Membrane Repair

A well-characterized assay (Bansal et al. 2003) was used to assess membrane repair. Membrane repair is initiated by creating a lesion of a well-defined size and shape (software selectable) in cells immersed in the dye, FM1-43. Previous studies have demonstrated that lesions of a defined size can reproducibly be made using this technique (Bansal et al. 2003; Howard et al. 2011a, b). FM1-43 is non-fluorescent in water, but highly fluorescent in a non-polar environment, such as cell membranes. It is capable moreover of rapidly partitioning into and out of lipid bilayers, but cannot cross them. In the absence of a lesion, therefore, only the surface lipid bilayer, the plasma membrane, is labeled, as well as over time (hours) endosomal pathway membranes. Over the time course of the typical repair experiment, this endocytotic accumulation of fluorescence is however insignificant (Bansal et al. 2003; Howard et al. 2011a; b). When a membrane lesion is created with the laser, dye in medium can freely enter the cell, where it then partitions into internal membrane compartments, adding internal fluorescence signal. Accumulation of this signal continues until repair is completed. Continuing accumulation of internal fluorescence, recorded by measuring integrated cellular fluorescence signal over time, therefore provides an accurate record of the duration of lesion opening, and the cessation of dye accumulation marks repair completion. Briefly, cultured cells were wounded in PBS, with or without 1.2 mM Ca^2+^, containing 2.5 μM FM 1-43 (Invitrogen). Laser injury was produced using a 2-photon laser scanning confocal microscope (LSM 780 Multiphoton Microscope, Zeiss) coupled to a Vision S tunable laser (Coherent) at 100 % power (one laser iteration and a 15 pixels diameter circle bleach area placed over the membrane edge), creating 1.66 µm diameter plasma membrane disruptions. Fluorescence intensity over time was quantified using ZEN 2012, Zeiss software.

### Viability After Electroporation (With or Without Added Calcium)

The primary normal cell line (HDF-n) and three cancer cell lines (HT29, MDA-MB231, and SW780) were tested for viability after electroporation. These cell lines were chosen to compare the normal cell line with a few of the cancer cells lines that had shown a clear difference in membrane repair.

After harvesting, cells were washed in HEPES buffer (10 mM HEPES, 250 mM sucrose, and 1 mM MgCl_2_ in sterile water) and diluted to 5.5 × 10^6^ cells/ml HEPES buffer. In 4 mm cuvettes with aluminum electrodes (Molecular BioProducts, Inc.) 300 µl cooled cells (8 °C) were electroporated by delivering 8 pulses of 100 µs, 1.2 kV/cm, and 1 Hz using a BTX T820 square wave electroporator. After 20 min incubation at 37 °C and 5 % CO_2_ cells were diluted in culture medium to 3.1 × 10^5^ cells/ml and seeded in 96-well plates (100 µl/well). One day after treatment viability was measured by MTS assay (Malich et al. 1997) using Multiskan-Ascent ELISA reader (Thermo Labsystems).

### Permeabilization After Electroporation

The primary normal cell line (HDF-n) and the bladder cancer cell line (SW780) were tested for degree of permeabilization after electroporation. These cell lines were chosen since they showed a significant difference in viability after electroporation.

**Fig. 2:**
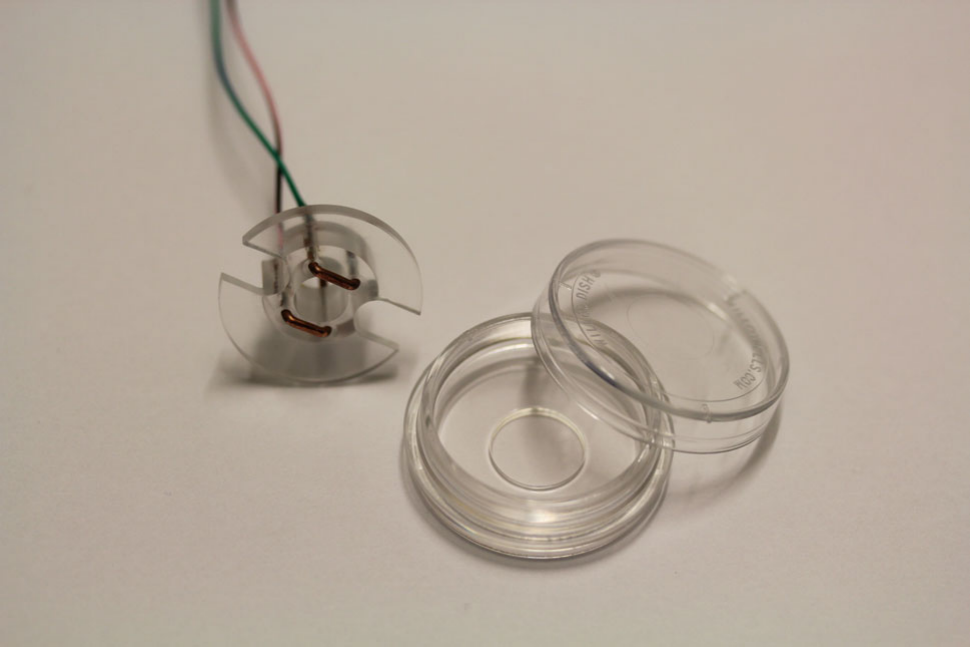
Custom-made contact copper electrode. Picture of the electrode used for the experiment testing permeabilization after electroporation. The electrode is made to fit in a Willco Well

Cells were plated in Willco wells (30.000 cells in 2 ml medium; WillCo Wells BV, The Netherlands) one day prior to experiments. Cells were washed in 1 ml Krebs–Ringer buffer (25 mM Na-gluconate, 120 mM NaCl, 1 mM MgCl_2_·6H_2_O, 0.4 mM KH_2_PO_4_, 1.6 mM K_2_HPO_4_·3H_2_O, 1.5 mM CaCl_2_·2H_2_O, 10 mM glucose·H_2_O in MiliQ water) before adding 400 µl Krebs–Ringer buffer containing 1 µM YO-PRO-1 (Invitrogen) and incubated at 37 °C and 0 % CO_2_ for 30 min. YO-PRO-1 is a fluorescent dye that binds to nucleic acids after entering cells when plasma cell membranes are permeabilized (e.g., electroporation, during apoptosis, induction of pore channels). Cells were treated with electroporation (8 pulses of 1.2 kV/cm, 100 µs, and 1 Hz) using a Cliniporator (IGEA, Italy) and a custom-made contact copper electrode with 8 mm between the electrodes (Fig. 2). Electroporation parameters were optimized for both cell lines for high permeabilization and high viability, and the same parameters were used for both cell lines. Pictures were taken before treatment and 3 min after treatment using a Leica DMI6000B microscope connected to a Leica DFC450C camera. Mean fluorescence intensity in cells was calculated using Image J software (NIH, Bethesda, USA).

### Graphics and Statistics

All artwork was created using GraphPad Prism 6. Statistical analyses were performed using SAS software (version 9.2). Difference in fluorescence intensity in the different cell lines was evaluated as repeated measurements, validated and analyzed with an exponential decrease model with Bonferroni correction. “Cell line,” “Time,” and “n” were used as factors and baseline level of fluorescence intensity were used as covariant. Difference in viability after electroporation between different cell lines and difference in permeabilization after electroporation between HDF-n and SW780 cell lines were assessed using one-way analysis of variance (ANOVA) with Bonferroni correction. Difference in viability after electroporation with addition of calcium between HDF-n and HT29 cell lines was assessed using two-way ANOVA with Bonferroni correction.

## Results and Discussion

**Fig. 3:**
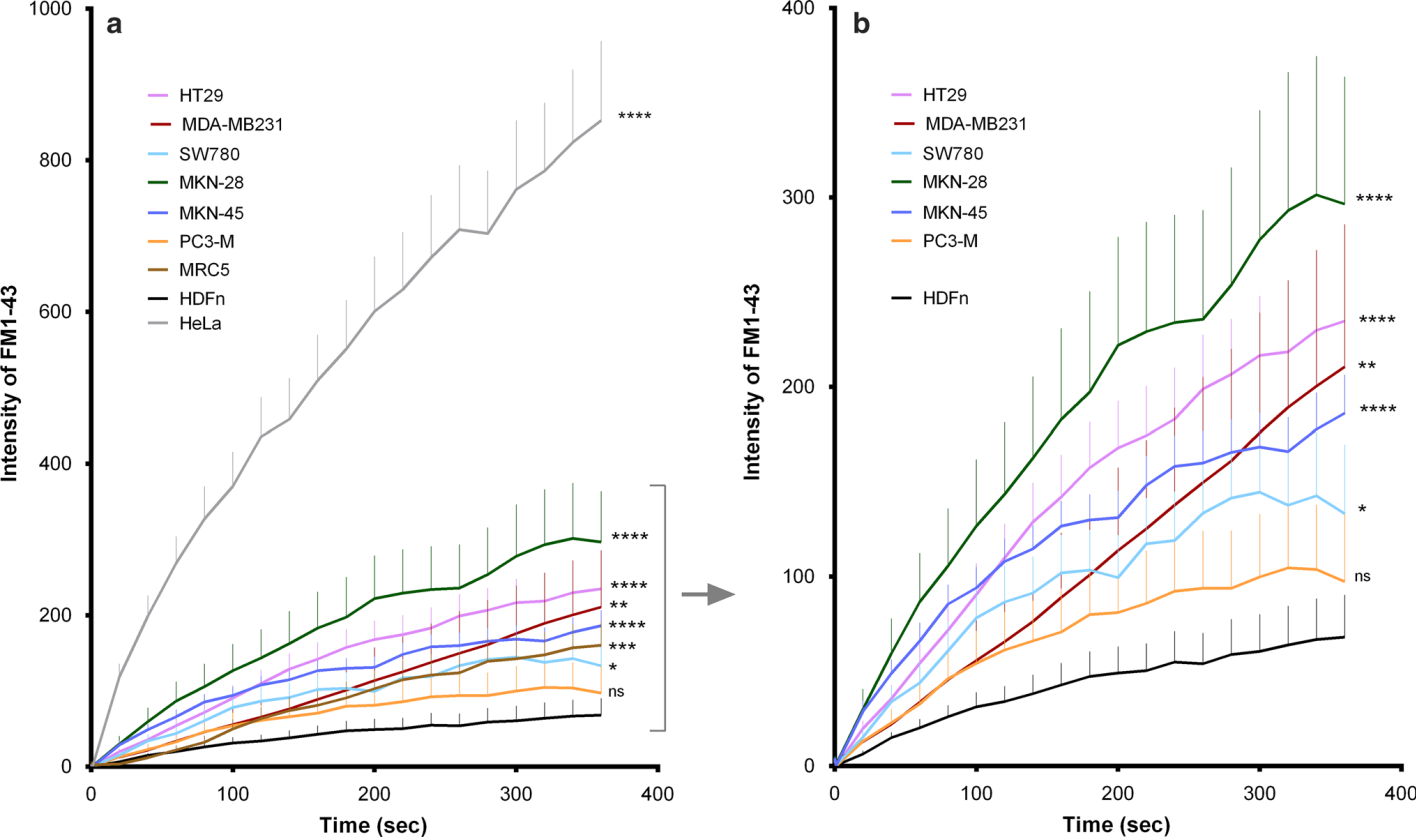
Membrane repair capability. Intensity of FM1-43 in nine different cell lines (seven cancer cell lines, an immortalized cell line, and a normal primary cell line) after disrupting the plasma membrane using laser. **a** All tested cell lines including the immortalized cell line and the HeLa cell line. **b** Six of the tested cancer cell lines and the normal primary cell line, (note the changed *y*-axis). Data are shown as mean + SEM, *n* = 7–22, significance at 360 s shown, *p < 0.05, **p < 0.01, ***p < 0.001, ****p < 0.0001, *ns* not significant

**Fig. 4:**
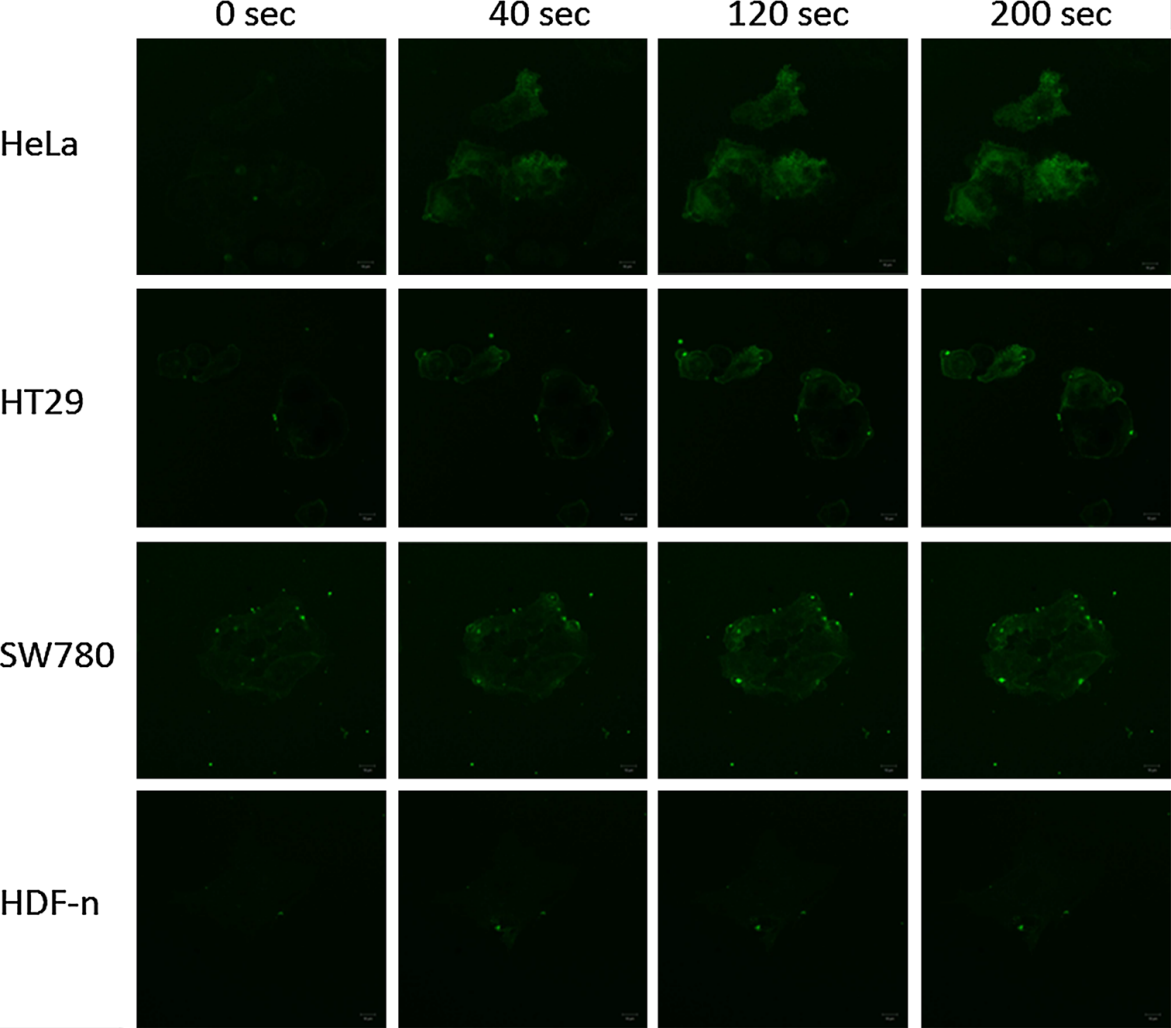
Images of FM1-43 intensity. Representative images of three cancer cells (HeLa, HT29, SW780) and a normal primary cell (HDF-n) showing fluorescence before and 40, 120, and 200 s after disrupting the plasma membrane using a laser in the presence of FM1-43. Scale bar in bottom right corner is 10 µm

To compare membrane repair in different cancer cell lines, an immortalized cell line, and a normal primary cell line, we measured the rate and extent of fluorescent dye entry after rupture of the plasma membrane using a laser (Figs. 3, 4). Nine different cell lines (seven cancer cell lines, an immortalized cell line, and a normal primary cell line) were tested to investigate membrane repair in a variety of different tumor types. The method used for testing membrane repair is a well-known method where a disruption in the plasma membrane is created using a laser (Bansal et al. 2003). This creates 1.66 µm diameter plasma membrane disruptions, and the size of the disruption does not depend on the cell type. The fluorescent dye enters through the membrane disruption, but further entry is hindered when plasma membrane repair is initiated. Prior dye entry by endocytosis into cytoplasm is ignored (subtraction of signal during analysis) and entry during the repair measurement is insignificant (second time scale). The rate and extent of dye entry in the normal primary cells was the lowest of all tested cell lines and significantly reduced compared to all the tumor-derived lines (*p* < 0.05) with one exception being the PC3-M cell line derived from a bone metastasis of a prostate cancer (*p* = 0.29) (Fig. 3). In five of seven cancer-derived cell lines, dye entry continued throughout the time course of the experiment (360 s). Thus, membrane repair either failed or was less effective in these tumor-derived cell lines indicating a less efficient membrane repair system. Especially HeLa cells have a much less effective membrane repair than all the other tested cell lines (Figs. 3a, 4). The immortalized normal cell line (MRC5, a human lung cell line) showed a slightly higher dye entry than the primary normal cell line (Fig. 3a). However, dye entry in the immortalized cell line was still in the lower half of the tested cell lines. Possible changes in membrane composition and/or membrane function when immortalizing the cell line may explain why this cell line does not exhibit dye entry equivalent to the primary normal cells. However, further investigations are needed to clarify if normal and immortalized cell lines in general show difference in membrane repair.

As previously described, permeabilization induced by electroporation depends on the cell type (membrane composition, cell size, and cell shape) (Teissie and Rols 1993; Pucihar et al. 2006; Levine and Vernier 2012). Differences in viability after electroporation have previously been explained by differences in permeabilization due to the different cell types. However, this study suggests that differences in membrane repair after permeabilization might also affect the viability. Electroporation induces permeabilization of the plasma membrane with more but smaller pores (Gehl 2003; Levine and Vernier 2012) compared with laser disruption, and this might lead to different repair mechanisms in the two cases.

**Fig. 5:**
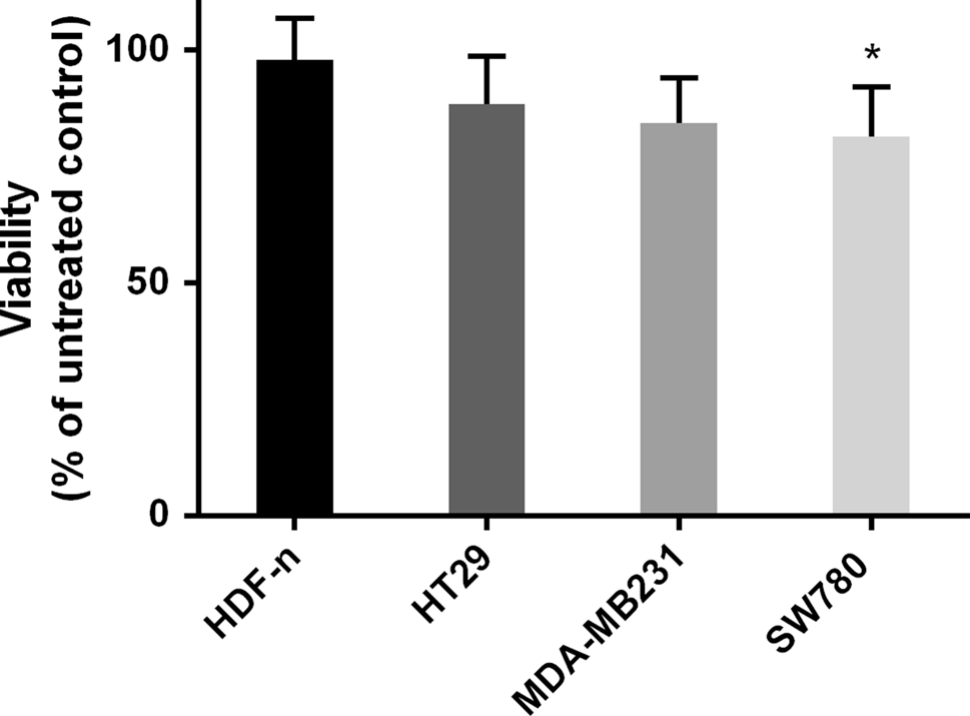
Viability after electroporation. Viability measured using MTS assay one day after electroporation (8 pulses of 1.2 kV/cm, 100 µs, and 1 Hz) of three cancer cell lines (HT29, MDA-MB231, and SW780) and a normal primary cell line (HDF-n). Data are shown as mean + SD *n* = 3–6, *p < 0.05

**Fig. 6:**
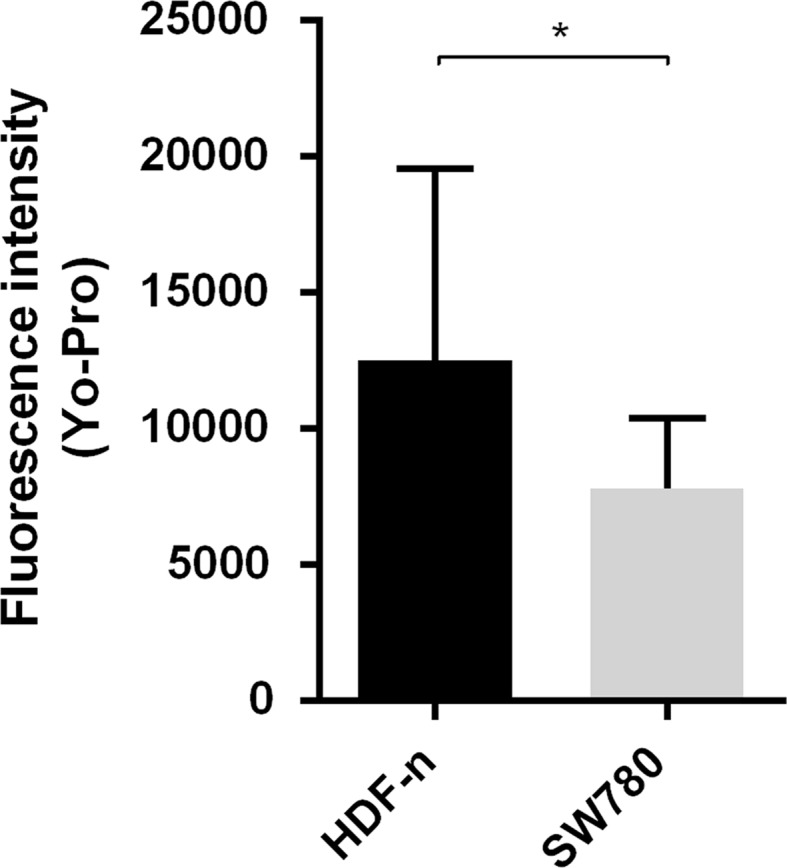
Permeabilization after electroporation. A normal primary cell line (HDF-n) and a bladder cancer cell line (SW780) electroporated in the presence of the non-permeant dye Yo-Pro-1. Fluorescence intensity was measured 3 min after electroporation. Data are shown as mean + SD *n* = 4, *p < 0.05

To test if this difference in membrane repair has an effect on viability when permeabilizing the plasma membrane by electroporation, we electroporated four of the used cell lines (three cancer cell lines and the normal primary cell line, previously used in another study (Frandsen et al. 2015)) and measured viability one day after treatment (Fig. 5). The normal primary cells showed the highest viability (98 %) after electroporation, significantly higher than viability of the SW780 cancer cell line (81 %, *p* < 0.05). This difference in viability after electroporation in the normal primary cell lines and the SW780 cancer cell line was not caused by lower permeabilization after electroporation of the normal cells. Actually, when testing permeabilization after electroporation in the two cell lines in the presence of the fluorescent dye YO-PRO-1, we showed that uptake of the dye was significantly higher in the normal cell line than in the cancer cell line. This indicates higher degree of permeabilization of the normal cell line after electroporation, when using the same electroporation parameters for both cell lines (Fig. 6). In other words, the normal cell line does get permeabilized by electroporation (even to a higher extent than the malignant cell line tested), but repairs faster when a direct comparison is made as the laser holes are comparable across cell lines. Thus, normal cells seem to recover more effectively, likely explaining the higher survival rate (Fig. 5). Survival after electroporation is determined by a number of factors, including the degree of membrane permeabilization, but also energy level and other intracellular factors. As seen in Fig. 3, there is a significant difference in membrane repair between normal and malignant cell lines, which may in part explain the difference in survival after electroporation. However, a more pronounced difference of late membrane repair would ensue when drugs (such as bleomycin) were added.

The results of this study indicate that there is a reduced ability of membrane repair in cancer cells compared with the normal cells. This might contribute to the difference in survival and effectivity of treatment on normal and cancer cells and tissues when using permeabilization methods as reported earlier (Lejbkowicz et al. 1993; Lejbkowicz and Salzberg 1997; Marty et al. 2006; Neal et al. 2011; Frandsen et al. 2015; Landstrom et al. 2015). Further investigations are needed. A possible difference in membrane repair might be caused by changes in the repair mechanisms and/or membrane composition in cancer cells. Membrane repair is a very complex system including several mechanisms and involving numerous proteins (Boucher and Mandato 2015). Many different Ca^2+^-sensors are involved in membrane repair including calpains, annexins, and S100 proteins. These sensors are activated by the high Ca^2+^ entry at the site of the injured membrane and initiate the membrane repair process (McNeil et al. 2006; Jaiswal et al. 2014; Boucher and Mandato 2015). Changes in the expression of these proteins might change the membrane repair mechanisms. Interestingly, expression of S100 and annexin proteins has been shown to be changed in many different cancer types (Bresnick et al. 2015; Wei et al. 2015). Thus, it would be relevant to make further investigation on the expression of these proteins in the 9 cell lines used in this study in order to test a possible correlation with the shown membrane repair results. The changed composition of lipids in the plasma membrane has also been shown in cancer cells, such as more negative charge, elevated levels of cholesterol, and the presence of certain lipids in the outer and inner leaflet (Zwaal et al. 2005; Schweizer 2009). A changed membrane composition might also affect membrane repair.

When treating cutaneous metastases with electrochemotherapy in the clinic, the surrounding normal tissue is less affected than the tumor tissue (Fig. 1) and it has been proposed to be due to the increased effect of chemotherapeutic drugs on fast-dividing cells as well as an increased conductivity in tumor tissue increasing the permeabilization (Mir et al. 1996; Laufer et al. 2010). The present study may supplement the previous explanations on the clear difference in sensitivity of cancer and normal cells treated with electrochemotherapy (Marty et al. 2006), as well as after calcium electroporation (Frandsen et al. 2015), other electroporation-based therapies (Neal et al. 2011), and other therapies causing membrane permeabilization such as sonoporation-based therapies (Lejbkowicz et al. 1993; Lejbkowicz and Salzberg 1997) as well as ionizing radiation (Hannig et al. 2000).

## Conclusion

In conclusion, membrane repair was less effective in six of the seven tested cancer cell lines and the immortalized cell line compared with the normal primary fibroblasts. This result could be part of the explanation why electroporation-based therapies and other therapies permeabilizing the plasma membrane are more effective in inducing cell death of malignant than normal cells. However, further investigations are needed to substantiate these results and investigate in more detail about membrane repair mechanisms and membrane composition in the different cell lines.
